# The mechanistic study of injectable hydrogel loaded with BMSC-exosomes in regulating the TGF-β/MMP axis to inhibit experimental myopia model

**DOI:** 10.1016/j.mtbio.2026.103051

**Published:** 2026-03-24

**Authors:** Jingwen Hui, Xiongfeng Nie, Zheya Han, Yuhua Rui, Yuxi Bai, Jingxuan Geng, Wenguang Liu, Quanhong Han

**Affiliations:** a Nankai University Optometry and Vision Science Institute, Nankai University Affiliated Tianjin Eye Hospital, Tianjin, 300071, China; b Tianjin Eye Hospital, Tianjin Key Laboratory of Ophthalmology and Visual Science, Tianjin Eye Institute, Tianjin, 300020, China; c School of Material Science and Engineering, Tianjin Key Laboratory of Composite and Functional Materials, Tianjin University, Tianjin, 300350, China

**Keywords:** Myopia, Exosomes, Injectable hydrogel, Scleral remodeling, Anti-inflammation

## Abstract

Pathological myopia involves reduced collagen deposition and scleral thinning, leading to axial elongation—a process exacerbated by chronic inflammation that disrupts scleral extracellular matrix homeostasis. To address this, we developed an injectable microgel system based on methacrylated alginate (AlgMA) loaded with BMSC-derived exosomes for preventing myopia progression. The photocrosslinkable AlgMA hydrogel exhibited a compressive modulus of ∼10 kPa, providing mechanical support for scleral reinforcement, while its porous network facilitated sustained exosome release. Processing into microgels (AlgMA-MGs) via repeated extrusion through a 25-gauge needle significantly improved injectability of AlgMA hydrogel. Exosome-loaded AlgMA-MGs (Exos-AlgMA-MGs) enhanced fibroblast proliferation and migration, downregulated MMP-2, and upregulated collagen I synthesis via modulation of the TGF-β/MMP axis. In a guinea pig form-deprivation model, both AlgMA-MGs and Exos-AlgMA-MGs attenuated axial elongation in myopic eyes, with the Exos-AlgMA-MGs showing optimal efficacy over four weeks, attributable to the combined biomechanical and bioactive effects. Histological analysis further confirmed that Exos-AlgMA-MGs increased scleral thickness and downregulated the expression of inflammatory factors in myopic eyes, thereby achieving effective anti-scleral remodeling. These findings suggest that Exos-AlgMA-MGs represents a promising strategy for controlling myopia progression.

## Graphical abstract

**Figure:**
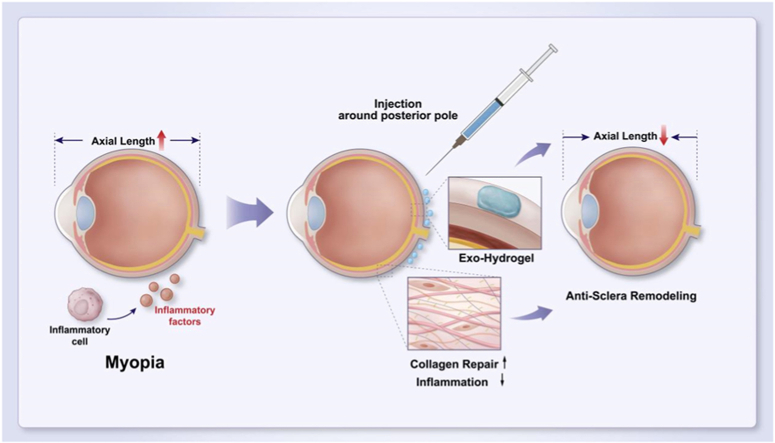

## 1 Introduction

Myopia is a globally prevalent refractive error characterized by distant images being focused in front of the retina, resulting in blurred distance vision [1,2]. In recent years, the incidence of high myopia (commonly defined as a refractive error of −6.00 diopters or more) has risen sharply worldwide, often accompanied by excessive axial elongation of the eyeball, leading to progressive structural changes at the posterior pole and ultimately the development of pathological myopia, characterized by posterior scleral thinning, retinal and choroidal atrophy, and, in some cases, posterior staphyloma [[3], [4], [5]]. In severe cases pathological myopia can result in complications such as retinal detachment, macular hemorrhage and choroidal neovascularization [6]. Because of its high prevalence and potential for irreversible vision loss, controlling the progression of myopia has become an important public health priority [7,8]. Posterior scleral reinforcement (PSR) is an effective surgical procedure aimed at controlling axial elongation to prevent the progression of pathologic myopia [9]. This technique involves the placement of reinforcing materials onto the posterior sclera to provide biomechanical support, thereby reducing axial extension of the sclera [10]. However, conventional PSR is an invasive surgery that demands precise surgical technique and extensive postoperative care [11]. Additionally, the procedure itself may trigger inflammatory responses and intraocular infections, among other complications [12].

Hydrogels, which exhibit physicochemical properties analogous to the natural extracellular matrix and demonstrate excellent biocompatibility, offer significant performance advantages over traditional PSR materials [[13], [14], [15]]. Consequently, they are attracting increasing attention and research interest in the field of myopia prevention [16,17] Notably, as a non-invasive and minimally invasive surgical approach, injectable hydrogels have emerged as a novel intervention strategy capable of replacing conventional PSR [18]. This technique can reduce surgical errors, shorten recovery time, and lower the risk of complications associated with larger incisions [19]. On the other hand, fibroblasts are responsible for producing collagen to maintain the extracellular matrix, thereby providing structural support to the sclera [20,21]. Compromised fibroblast function is a key factor in the pathogenesis of pathological myopia [20,22]. TGF-β signaling plays a central role in regulating collagen synthesis and matrix metalloproteinase activity in scleral fibroblasts, particularly MMP-2, which contributes to collagen degradation during myopia progression [21]. Fibroblast transplantation has been demonstrated to effectively control myopia progression [23]. In our previous study, a polysaccharide-based injectable hydrogel loaded with fibroblasts yielded a synergistic effect that combines cell therapy with physical reinforcement, resulting in improved control of axial elongation and facilitation of scleral physiological structure restoration [24,25]. Nevertheless, significant hurdles persist in fibroblast-based therapy, notably the long-term viability of post-implantation cells that demands further elucidation, along with its inherent immunogenicity [26].

In high myopia, the vitreous exists in a chronic, low-grade “para-inflammatory” state driven by mechanical stretch, choroidal thinning, and oxidative stress [8]. Bone marrow mesenchymal stem cells (BMSCs) have been widely utilized for regulating inflammatory microenvironments and promoting tissue repair, owing to their remarkable immunomodulatory properties [27]. Existing evidence indicates that the functional benefits of BMSCs are primarily attributed to their paracrine mechanisms, particularly mediated through the release of exosomes [28]. Exosomes are nanoscale extracellular vesicles, approximately 30–200 nm in diameter, which are enriched with various bioactive components such as proteins, lipids, and miRNAs [29]. They facilitate intercellular communication and convey the anti-inflammatory properties of BMSCs [30,31]. Given these characteristics, BMSC-derived exosome therapy for high myopia is emerging as a cell-free strategy to rebalance posterior pole remodeling by delivering bioactive microRNAs and proteins that temper inflammation and normalize extracellular-matrix turnover. Compared with cell transplantation, exosomes are less immunogenic and easier to standardize, while retaining potent paracrine effects [32]. Practical delivery favors sub-Tenon routes for scleral targeting, embedding exosomes in injectable hydrogels enables sustained, posteriorly localized release and higher on-target exposure. Alginate-based hydrogels, characterized by their favorable biocompatibility and injectability, have found extensive application in the field of ophthalmology, which makes them ideal carriers for exosome delivery [[33], [34], [35]].

**Scheme 1:**
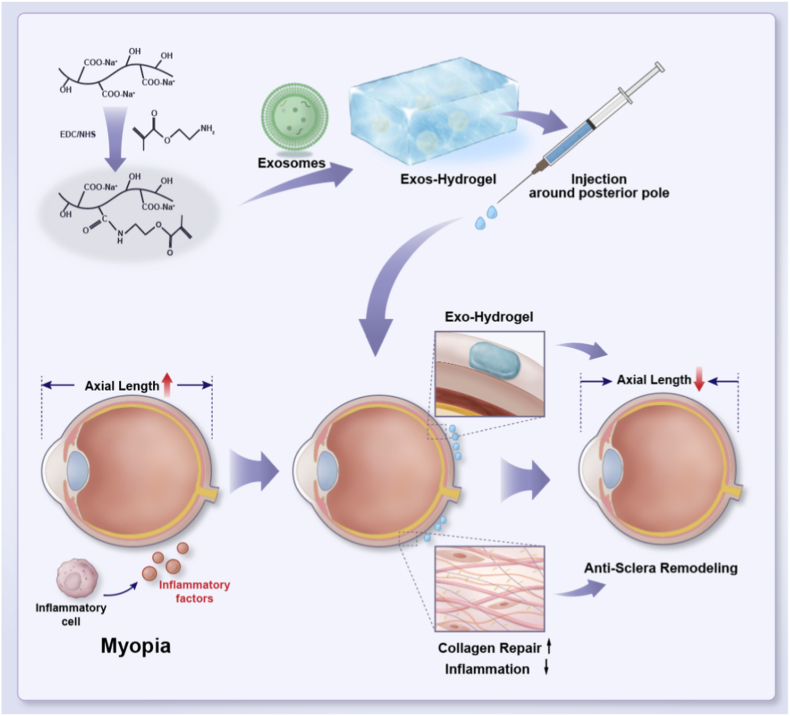
The mechanism of injectable exosome-loaded hydrogel for sclera remodeling to prevent development of myopia.

In this work, we developed an injectable alginate-based hydrogel loaded with BMSC-derived exosomes in regulating the TGF-β/MMP axis to inhibit experimental myopia model (Scheme 1). Photocrosslinkable methacrylated alginate (AlgMA) was successfully synthesized through the amidation reaction between alginate and 2-aminoethyl methacrylate hydrochloride. The porous structure of the AlgMA hydrogel facilitated a sustained release profile of the encapsulated exosomes. To enhance injectability, the AlgMA hydrogel was converted into microgels (AlgMA-MGs) through a process of repeated extrusion using a 25-gauge needle. In vitro cell experiments demonstrated that the exosomes released from AlgMA-MGs promoted fibroblast proliferation and migration, as well as increased the secretion of collagen type I (Col-I). Furthermore, the exosome-loaded hydrogel downregulated the expression of matrix metalloproteinase-2 (MMP-2). In the in vivo study, both hydrogel alone and exosome-loaded hydrogel effectively shortened axial length in the guinea pig form-deprivation model. The exosome-loaded formulation showed the greatest benefit, likely through the combined effects of exosomal bioactivity and mechanical support, maintaining axial shortening across the 4-week observation period. By enhancing collagen deposition, treatment was associated with increased scleral thickness and improved its biomechanical strength, helping restore normal scleral architecture. Together, these results indicate that an exosome-loaded injectable hydrogel may be a promising clinical strategy for controlling myopia progression.

## 2 Experimental section

### 2.1 Materials

Sodium alginate (Alg, low viscosity) was purchased from Alfa-Aesar. 1-ethyl-3-(3-dimethylaminopropyl) carbodiimide hydrochloride (EDC), N-hydroxysuccinimide (NHS) were purchased from Beijing InnoChem Technology Co.,Ltd. 2-Aminoethyl methacrylate hydrochloride (AEMA) was purchased from Shanghai Macklin Biochemical Co., Ltd. Bone Marrow-Derived Mesenchymal Stem Cell Exosomes were purchased from Cyagen Bioscience Inc.

### 2.2 Synthesis of AlgMA

Briefly, 6 g of sodium alginate was dissolved in 600 mL of deionized water. Subsequently, 6 g of EDC, 3.44 g of NHS, and 1.44 g of AEMA were added sequentially to the solution. The reaction was allowed to proceed under constant stirring at 25 °C for 24 h. The resulting mixture was then dialyzed (using a dialysis membrane with a molecular weight cutoff of 8 kDa) against deionized water for 3 days. Finally, the AlgMA product was obtained by freeze-drying.

The successful synthesis of AlgMA was confirmed by ^1^H NMR spectrum, using deuterium oxide (D_2_O) as the solvent.

### 2.3 Characterization of exosomes

The morphology of the exosomes was observed by transmission electron microscopy (TEM). Briefly, a diluted exosome solution was dropped onto a copper grid, air-dried at room temperature, and then imaged under TEM. Dynamic light scattering (DLS) was used to measure the particle size of exosomes. Exosome surface markers were analyzed using a bead-assisted flow cytometry method. Briefly, exosome suspensions were incubated overnight at 4 °C with aldehyde/sulfate latex beads (4 μm) under gentle rotation. Bead-bound exosomes were washed, incubated with FITC-conjugated anti-CD9 or anti-CD63 antibodies for 60 min at room temperature in the dark, and washed again prior to acquisition. Samples were analyzed on a BD FACSCanto II cytometer. Bead populations were gated based on forward and side scatter properties, and positive gates were defined using unstained controls. Results were expressed as the percentage of CD9^−^or CD63-positive events.

### 2.4 Preparation of AlgMA, Exos-AlgMA and Exos-AlgMA-MGs hydrogel

The AlgMA hydrogels were fabricated via UV-induced photocrosslinking. First, a sterile 3 w/v % AlgMA solution was prepared in PBS. Then, LAP was added to the solution to achieve a final concentration of 0.25 wt %. Finally, the precursor solution was irradiated with 365 nm UV light at a predetermined intensity for 150 s to form the AlgMA hydrogel. For the preparation of exosome-loaded AlgMA hydrogels (Exos-AlgMA), AlgMA was first dissolved in a PBS solution of exosomes (25 μg/mL), which was subsequently subjected to the photocrosslinking process.

To enhance injectability for in vivo applications, bulk AlgMA and Exos-AlgMA hydrogels were processed into microgels (AlgMA-MGs or Exos-AlgMA-MGs) via five cycles of extrusion through a 25-gauge needle.

### 2.5 Mechanical properties

The compressive properties of cylindrical AlgMA and Exos-AlgMA hydrogels (diameter: 5 mm; height: 5 mm) were tested at room temperature using an Instron 2344 Microester system. The compressive modulus was determined from the slope of the linear region (10% to 20% strain) of the stress-strain curve. A movement speed of 10 mm/min was applied during the tests.

### 2.6 Microscopic morphology

The microstructure of the hydrogel was characterized using scanning electron microscopy (SEM, Hitachi SU1510). The samples were rapidly frozen in liquid nitrogen and subsequently dehydrated in a freeze-dryer. The freeze-dried hydrogels were fractured to expose the internal cross-section, which was then sputter-coated with gold for 60 s before SEM observation.

### 2.7 Release of exosomes from hydrogels

To measure the release of exosomes from the AlgMA hydrogel, the Exos-AlgMA hydrogel was immersed in PBS and incubated at 37 °C for 10 days. The PBS solution was replaced daily, and the cumulative release of exosomes was determined using a Micro BCA assay kit (Boster, Wuhan, China).

Micro BCA assay:BSA standard solutions at graded concentrations and the BCA working solution were prepared according to the protocol. Subsequently, 150 μL of each standard and test sample were added into a microplate. An equal volume (150 μL) of the BCA working solution was added to each well and mixed thoroughly for 30 s. The microplate was incubated at 60 °C for 1 h. After cooling to room temperature, the absorbance of each well was measured at 562 nm within 10 min using a microplate reader. The concentration of released BSA in the samples was calculated based on a standard curve generated from the BSA standard solutions.

### 2.8 Rheological properties

The rheological properties of the hydrogels were characterized using a rheometer (MCR302, Anton Paar) equipped with a 25-mm parallel plate geometry. The measuring gap was set to 0.8 mm. An adequate amount of deionized water was placed around the measuring system to prevent water evaporation from the hydrogel samples. The thermosensitive behavior was evaluated by monitoring the storage modulus (G′) and loss modulus (G″) of both AlgMA and AlgMA-MGs as the temperature increased from 20 °C to 50 °C. To evaluate the mechanical stability, the hydrogels were subjected to frequency sweeps from 0.1 to 10 Hz, during which G′ and G″ were monitored. To evaluate the injectability of the hydrogels, strain sweep and shear thinning tests were conducted. The variations in the G′ and G″ were recorded over a strain range of 1% to 1000%, and the viscosity was measured as a function of shear rate from 1 to 1000 s^−1^. To investigate the thixotropic recovery and strain-dependent viscoelasticity, measurements were performed under an alternating step strain protocol (1%-50%-1%-100%-1%) at a fixed frequency of 1 Hz, recording the evolution of G′, G″, and viscosity.

### 2.9 Subcutaneous implantation and histological analysis

Male C57BL/6 mice (6 weeks old, 20 g) were used to evaluate the inflammatory response after the hydrogel implantation. The mice were anesthetized using inhaled anesthesia. A 60 μL AlgMA-MGs hydrogel was subcutaneously injected into the dorsal region of each mouse using a 1 mL syringe. At weekly intervals, mice were euthanized to examine the retention of the hydrogel at the implantation site and the associated inflammatory response. Furthermore, tissue surrounding the hydrogel retrieved at the 4-week time point was subjected to hematoxylin and eosin (H&E) staining for histological analysis.

### 2.10 In vitro cell experiments

#### 2.10.1 Culture of fibroblasts

Mouse fibroblast cells were obtained from BFB Company (Shanghai, China). The cells were cultured in complete medium consisting of Dulbecco's Modified Eagle Medium (DMEM) supplemented with 10% fetal bovine serum (FBS) and 1% penicillin/streptomycin. All cultures were incubated at 37 °C in a humidified atmosphere containing 5% CO_2_. Upon reaching 80% confluency, the cells were passaged or harvested using 0.25% trypsin.

#### 2.10.2 Cell proliferation assay

The effects of AlgMA-MGs, exosomes, and Exos-AlgMA-MGs on fibroblast proliferation were evaluated. Four different cell media were prepared: (1) serum-free medium (SFM); (2) an extract of AlgMA-MGs in SFM (hydrogel mass concentration: 100 mg/mL); (3) SFM containing exosomes (2.5 μg/mL); and (4) an extract of Exos-AlgMA-MGs in SFM (hydrogel mass concentration: 100 mg/mL). Fibroblasts were suspended in complete medium, seeded into cell culture plates, and cultured for 24 h. The complete medium was then aspirated and replaced with the four test media, followed by a 48-h incubation period. Cell viability was assessed using live/dead staining and CCK-8 assays at the 24 h and 48 h time points.

#### 2.10.3 Scratch assay for fibroblast migration

The effect of the four cell media on fibroblast migration was evaluated using a scratch assay. Fibroblasts were first cultured in complete medium until they reached 100% confluency. A linear scratch was then created in the cell monolayer using a sterile 10 μL pipette tip, followed by washing with PBS to remove dislodged cells. The complete medium was replaced with the four cell media, and the cells were further incubated for 48 h. Microscopic images of the scratch were captured at 0h, 24 h and 48 h to assess fibroblast migration into the wound area. The relative wound healing rate was quantified based on the image analysis.

#### 2.10.4 Expression of Col Ⅰ and MMP-2

To evaluate the influence of the four media on Col I and MMP-2 protein expression, fibroblasts were first allowed to adhere in complete medium for 24 h. The cells were then treated with the respective test media for another 24 h. Subsequently, the culture supernatant was centrifuged and analyzed using specific enzyme-linked immunosorbent assay (ELISA) kits.

ELISA assay of Col Ⅰ:The cell medium was centrifuged at 3000×*g* for 10 min to collect the supernatant. After preparing standard solutions, 50 μL of standards or samples were added to the microplate. The plate was incubated at 37 °C for 30 min, washed, and then 50 μL of enzyme conjugate was added. Following another incubation and wash, 50 μL each of Chromogen A and B were added for 10-min color development at 37 °C in the dark. The reaction was stopped with 50 μL stop solution, and the absorbance was measured at 450 nm. Sample concentrations were determined from the standard curve.

Similarly, the expression of MMP-2 protein in fibroblasts under different culture conditions was analyzed using a MMP-2 ELISA kit, following the manufacturer's instructions.

### 2.11 Animal experiments

#### 2.11.1 Form deprivation and exosome-loaded hydrogel transplantation

All experimental protocols were approved by the Ethics Committee of Tianjin Eye Hospital and conformed to the NIH/National Research Council Guide for the Care and Use of Laboratory Animals. Before any interventions, animals were randomized using a random-number table into six groups (n = 10 per group). Group assignments were concealed during the treatment phase to minimize selection bias. Animals were housed in plastic cages with wire lids under a 12-h light/dark cycle, with ad libitum access to water and daily vegetables.

Form deprivation-induced myopia was established in 3-week-old guinea pigs by applying a cream balloon occluder to the right eye and maintaining continuous deprivation for 28 days. After completion of the form deprivation period, animals received posterior scleral injection according to their assigned treatment groups, with the aim of evaluating therapeutic effects on established myopic scleral remodeling rather than preventive intervention during myopia development. Under an operating microscope, guinea pigs underwent posterior sub-Tenon's injection at the posterior pole under inhalational anesthesia. A single posterior scleral injection was performed for each animal immediately after completion of the 28-day form deprivation period, and no repeated administrations were conducted during the subsequent monitoring period. An injectable AlgMA-based hydrogel, with or without encapsulated exosomes, or control solutions were delivered into the sub-Tenon's space via the inferotemporal quadrant using a 1-mL syringe fitted with a 25-gauge needle. All exosome-loaded hydrogels were freshly prepared on the day of surgery. A uniform volume of 40 μL was injected into the right eye, and no sutures were required. Postoperatively, topical gatifloxacin was administered once daily. No perioperative deaths occurred, and no intraoperative complications were observed.

To delineate group assignment and distinguish the effects of individual components, six experimental groups were established: (1) Normal control (no intervention); (2) Myopia model (form deprivation only); (3) M-Exosome (myopic guinea pigs receiving exosome suspension alone); (4) M-Hydrogel (hydrogel injection without exosomes); (5) M-Exosome-Hydrogel (exosome-loaded hydrogel injection); and (6) M-PBS (phosphate-buffered saline injection). Each group received a single posterior scleral injection after completion of the form deprivation period.

#### 2.11.2 Axial length measurements

For each group, axial length was measured at one of five time points (0, 1, 2, 3, or 4 weeks) using an MD-1000A ultrasonic biometer. All measurements were performed under inhalational anesthesia, and mydriasis was achieved with three instillations of 0.5% phenylephrine.

#### 2.11.3 Intraocular pressure measurements

Intraocular pressure (IOP) was recorded at baseline (prior to occlusion/surgery) and at weeks 1, 2, 3, and 4, matching the follow-up schedule used for axial biometry. A handheld rebound tonometer designed for small animals was used under light inhalational anesthesia (isoflurane 1.5–2.0% in oxygen at 0.8–1.0 L min^−1^) to minimize stress-related transients; no topical anesthetic was instilled to avoid pharmacologic effects on IOP. Animals were positioned supine with the head in neutral alignment. For each eye (right, treated/myopic; left, fellow control), six valid taps were acquired and automatically averaged by the device to yield one measurement; three such measurements separated by ≥ 30 s were obtained and averaged to produce the per-eye IOP.

#### 2.11.4 OCT imaging

Optical coherence tomography (OCT) was performed at baseline and at weeks 2 and 4. As in Sections 2.11.2, 2.11.3, light inhalational anesthesia was used, followed by pharmacologic mydriasis with three instillations of 0.5% phenylephrine at 5-min intervals. To maintain corneal clarity and reduce aberrations, sterile artificial tears were applied. A spectral-domain OCT (SD-OCT) system equipped with a small-animal imaging module acquired posterior-pole volume scans centered on the optic nerve head using a raster protocol (e.g., 512 A-scans × 25 B-scans). Each B-scan used 3-10 frame averaging to reduce speckle. Automated retinal-layer segmentation was reviewed and manually corrected by a masked grader when necessary. Full retinal thickness was defined as the distance from the internal limiting membrane to the outer border of the RPE/Bruch's complex. Within a 1.0-mm-radius ring centered on the optic nerve head (excluding the nerve head), three equidistant sampling loci (superior, inferior, temporal) were measured; each locus was averaged from three repeats, and the mean of the three loci yielded the eye-level central retinal thickness. Scans with signal strength <7/10 or with motion/optical artifacts were repeated or excluded.

#### 2.11.5 Histological assessment of the sclera

At week 4, animals were euthanized and eyes were enucleated and fixed in 4% paraformaldehyde. Globes were paraffin-embedded, sectioned at 5 μm, and stained with hematoxylin and eosin for routine histology. For immunofluorescence, sections were deparaffinized and rehydrated, incubated with anti–collagen I (Col I; 1:150, Abcam) followed by goat anti–mouse secondary antibody (1:200, Abcam), and counterstained with DAPI. H&E and Col I/DAPI images were captured using a whole-slide scanner (3DHISTECH P250 FLASH)

#### 2.11.6 Western blot analysis

At week 4, scleral tissues were homogenized in RIPA lysis buffer supplemented with protease and phosphatase inhibitors. Protein concentrations were determined using a BCA protein assay. Equal amounts of total protein were separated by SDS–PAGE and transferred onto PVDF membranes. After blocking with 5% non-fat milk in TBST, membranes were incubated overnight at 4 °C with primary antibodies against TGF-β1, phospho-Smad2/3 (p-Smad2/3), total Smad2/3, and β-actin (loading control). After washing, membranes were incubated with HRP-conjugated secondary antibodies at room temperature. Protein bands were visualized using enhanced chemiluminescence (ECL) and quantified by densitometric analysis. Relative protein expression levels were normalized to β-actin, and p-Smad2/3 levels were further normalized to total Smad2/3.

### 2.12 Statistical analyses

All analyses were conducted in SPSS Statistics 27.0. Between-group comparisons used one-way ANOVA, and longitudinal outcomes were analyzed with repeated-measures ANOVA to account for within-subject dependence, as appropriate to the data structure. When the assumption of homogeneity of variances was met, least significant difference (LSD) post hoc tests were applied. Data are reported as mean ± standard deviation (SD). Statistical significance was set at α = 0.05, with significance codes: ns (p > 0.05), ∗ (p < 0.05), ∗∗ (p < 0.01), and ∗∗∗ (p < 0.001). In addition to p-values, effect sizes (Cohen's d) and 95% confidence intervals (CIs) were provided to aid interpretation of statistical and clinical relevance.

## 3 Results

### 3.1 Preparation of AlgMA and Exos-AlgMA hydrogel

**Fig. 1:**
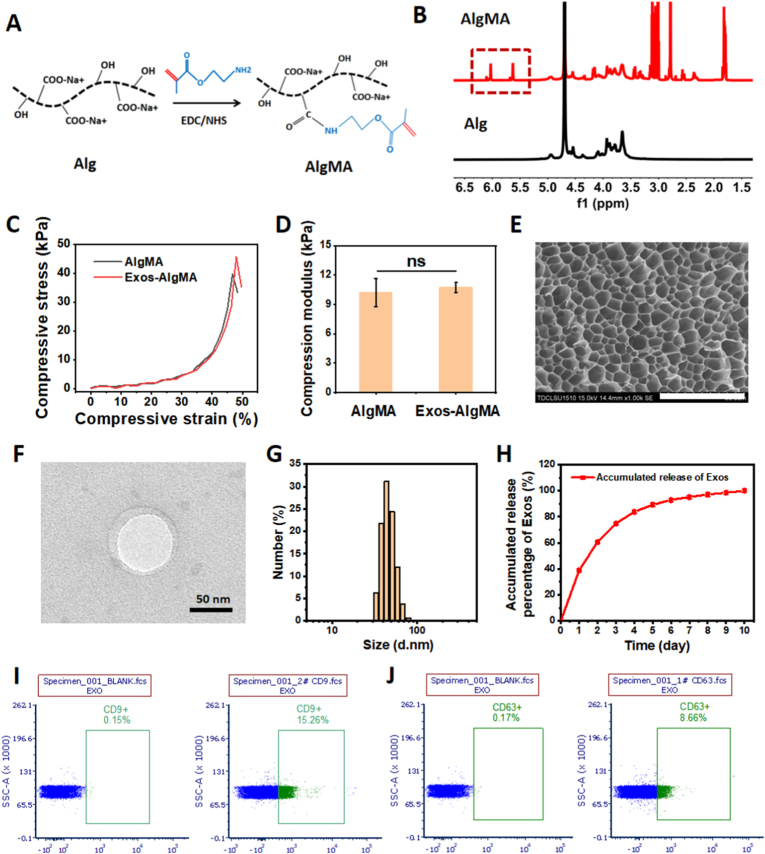
Synthesis, characterization, and exosomes release profile of Exos-AlgMA hydrogels. (A) Schematic diagram of the synthesis of AlgMA. (B) The ^1^H NMR spectra of Alg and AlgMA. (C) Compressive stress-strain curves and (D) corresponding compressive moduli of AlgMA and Exos-AlgMA hydrogel. (n = 3; ns: no significance). (E) SEM micrograph of the porous structure of the AlgMA hydrogel. Scale bar: 50 μm. (F) TEM image of BMSC-derived exosomes. Scale bar: 50 nm. (G) Particle size distribution of exosomes measured by DLS. (H) Cumulative release profiles of exosomes from the Exos-AlgMA hydrogel over 10 days. (I, J) Bead-assisted flow cytometry analysis of exosomal markers (CD9 and CD63). Percentages indicate CD9^+^ or CD63^+^ events within the bead gate, with unstained/blank controls used to define positive thresholds.

Here, the photocrosslinkable AlgMA hydrogel was selected as a carrier for exosomes owing to its excellent biocompatibility, favorable extrudability, and capacity for long-term retention in vivo. Synthesis of AlgMA was achieved by grafting methacrylate groups onto the alginate backbone through an EDC/NHS-catalyzed amidation reaction (Fig. 1A). The ^1^H NMR spectrum of AlgMA exhibited two new characteristic peaks at approximately 5.6 and 6.0 ppm, corresponding to the vinyl protons of the methacrylate group, which confirm its successful synthesis (Fig. 1B). The AlgMA solution rapidly photocrosslinked into a hydrogel within 150 s in the presence of the photoinitiator LAP, and the incorporation of BMSC-derived exosomes during this process yielded exosome-loaded AlgMA hydrogels (Exos-AlgMA). The compressive properties of the hydrogels were characterized. As shown in Fig. 1C and D, both AlgMA and Exos-AlgMA hydrogels exhibited similar stress-strain curves, with compressive moduli of 10.2 ± 1.4 kPa and 10.7 ± 0.5 kPa, respectively, showing no significant difference. This demonstrates that both hydrogels possess suitable mechanical strength for injection into the posterior sclera to provide physical support, thereby potentially mitigating axial elongation. Moreover, the Exos-AlgMA hydrogel offers an additional bioactive advantage through the sustained release of exosomes, which can modulate the inflammatory microenvironment of the sclera. As shown in Fig. 1E, SEM characterization revealed that the AlgMA hydrogel possessed a dense and uniform porous network. This microstructure is conducive to the sustained release of exosomes. Under TEM observation (Fig. 1F), BMSC-derived exosomes exhibited a characteristic cup-shaped morphology with a clearly discernible bilayer membrane structure and an average diameter of approximately 84 nm. The integrity of this membrane structure is essential for encapsulating bioactive cargo (e.g., proteins, nucleic acids) and maintaining their biological function [36,37]. In addition, DLS results showed that the particle size distribution of exosomes ranges from 32.7 to 91.3 nm (Fig. 1G), which falls within the standard size range for exosomes (30–200 nm). As shown in Fig. 1H, we evaluated the release of exosomes from the Exos-AlgMA hydrogel over a 10-day period in vitro. Notably, when the Exos-AlgMA hydrogel was immersed in a large volume of PBS at 37 °C, an initial burst release of approximately 74.8% of the loaded exosomes was observed within the first 2 days, followed by a sustained, gradual release over the subsequent 8 days. The posterior sclera, being an avascular and low-fluid environment, differs significantly from the in vitro release conditions. It is therefore reasonable to expect that the initial burst release would be attenuated, leading to a more extended release duration in practical applications. Exosome identity was further confirmed by bead-assisted flow cytometry. Compared with unstained/blank controls, bead-bound exosome samples exhibited increased FITC signals for CD9 and CD63, with CD9^+^ and CD63^+^ event percentages of 15.26% and 8.66%, respectively (Fig. 1I and J).

### 3.2 Preparation, injectability and biosafety of AlgMA-MGs hydrogel

**Fig. 2:**
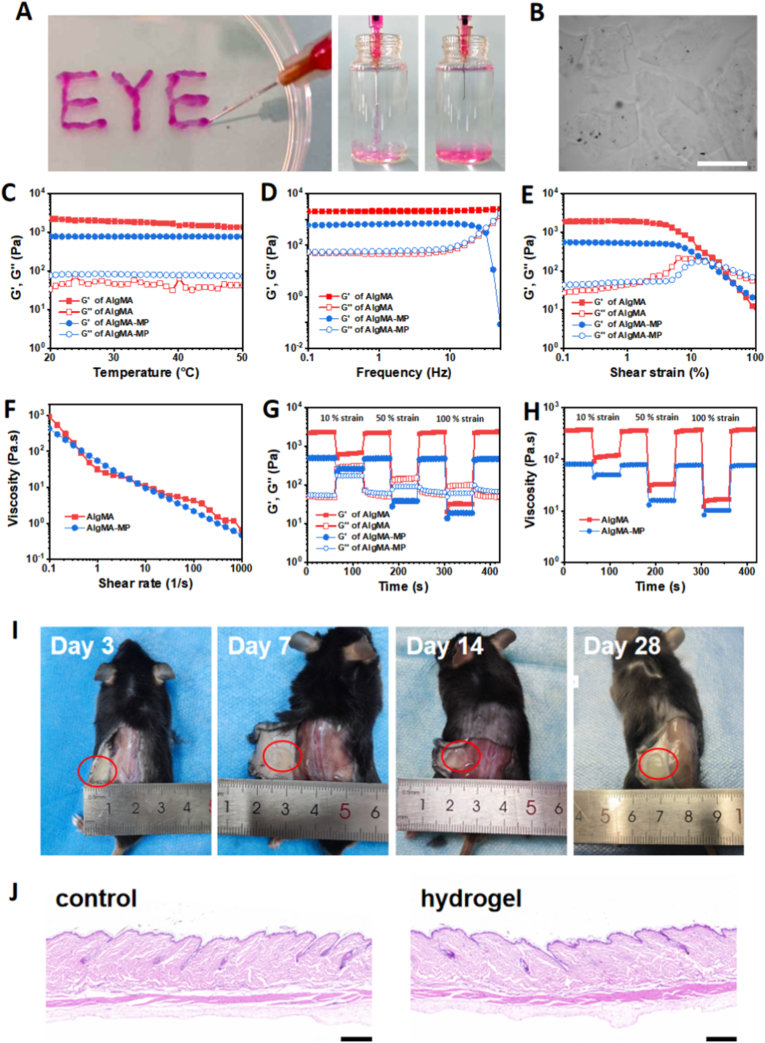
Characterization of injectability and biosafety of AlgMA-MGs. (A) Schematic of the injection process of AlgMA-MGs. (B) Microscopic photograph of AlgMA-MGs. Scale bar: 300 μm. (C) Temperature sweep of G′ and G″ for AlgMA and AlgMA-MGs from 20 °C to 50 °C. (D) Frequency dependence of G′ and G″ between 1 and 50 Hz. (E) Strain amplitude sweep from 0.1% to 100% strain. (F) Viscosity as a function of shear rate (0.1–1000 s^−1^). (G, H) Evolution of G′, G″, and viscosity during the oscillatory strain sweep (1%-10%-1%-50%-1%-100%-1%). (I) Subcutaneous implantation of AlgMA-MGs hydrogel within 4 weeks in vivo. (J) H&E-stained tissue section of the implantation site and normal tissue at week 4. Scale bars: 200 μm.

Alginate-based hydrogels exhibit outstanding rheological properties, including notable shear-thinning behavior, tunable viscoelasticity, and excellent self-healing capacity. These characteristics not only facilitate minimally invasive implantation via injection but also enable the maintenance of a stable three-dimensional microenvironment for cells or drugs in vivo, which is a key reason for their significant attention in the biomedical field. Herein, to improve the injectability of the photocrosslinked bulk AlgMA hydrogel, it was processed into microgels (AlgMA-MGs) by subjecting it to five extrusion cycles through a 25-gauge needle. The AlgMA-MGs demonstrated enhanced extrusion performance, allowing for continuous patterning as depicted in Fig. 2A. Injection of the AlgMA-MGs into PBS solution revealed their distinct microparticulate morphology, confirming the successful formation of microgels. Fig. 2B shows that the mechanically extruded AlgMA-MGs possessed an irregular polygonal microgel morphology and a size distribution under 300 μm.

The rheological properties of the AlgMA hydrogel and the AlgMA-MGs were characterized and compared. Temperature sweep tests revealed that neither the AlgMA nor the AlgMA-MGs exhibited significant thermosensitivity; both maintained a stable gel state with a G′ greater than the G″ throughout the 20–50 °C range (Fig. 2C). This indicates their capability to remain structurally stable at body temperature after implantation. The frequency sweep tests revealed that both AlgMA and AlgMA-MGs maintained a stable gel state (G' > G″) within the low-to-medium frequency range (1–10 Hz). However, at a higher frequency of 50 Hz, the AlgMA-MGs exhibited a crossover point where G″ surpassed G′, indicating a gel-sol transition (Fig. 2D). Strain amplitude sweeps demonstrated that both hydrogels possessed a well-defined linear viscoelastic region at low strains (0.1%–1%), with the gel-sol transition occurring at strains of approximately 16% and 27% for AlgMA and AlgMA-MGs, respectively (Fig. 2E). Furthermore, both AlgMA and AlgMA-MGs exhibit typical shear thinning behavior, with their viscosity significantly decreasing with increasing shear rate (Fig. 2F). Notably, the AlgMA-MGs displayed a lower viscosity under high shear rates compared to its bulk AlgMA hydrogel. We further assessed the thixotropy of the hydrogels through cyclic step strain measurements. As shown in Fig. 2G, when the shear strain increased from 1% to 50%, AlgMA-MGs immediately transitioned to a sol state, while AlgMA underwent a more gradual gel-sol transition. At a higher strain of 100%, both hydrogels rapidly transformed into a sol state, with AlgMA-MGs exhibiting lower G′ and G″ values than AlgMA. Upon returning to 1% strain, both materials quickly recovered their initial gel state (G' > G″), demonstrating excellent structural recovery. Throughout the cyclic strain step test, the viscosity of AlgMA-MGs also exhibited thixotropy and remained significantly lower than the value of AlgMA (Fig. 2H). The results indicate suitable injectability for both hydrogels. AlgMA-MGs demonstrate superior rheological performance characterized by a lower critical strain, reduced viscosity, and decreased modulus. These attributes minimize energy consumption during deformation, thereby offering lower injection resistance and enhanced flowability.

The AlgMA-MGs were subcutaneously injected into mice to assess its in vivo retention and biocompatibility. As shown in Fig. 2I, the AlgMA-MGs implant exhibited no significant degradation over the 28-day period and maintained long-term stability. Furthermore, no significant signs of inflammatory response were observed in the tissues following either short-term (3-day) or long-term (28-day) hydrogel implantation. Histological analysis was performed via H&E staining on skin tissue following 28-day implantation of AlgMA-MGs, with comparison to normal tissue. As shown in Fig. 2J, no observable inflammatory cell infiltration was detected in the hydrogel-implanted tissue compared to the normal control. These results collectively demonstrate the excellent biocompatibility of the AlgMA-MGs hydrogel in vivo, suggesting its promising biosafety as an ophthalmic implant material.

### 3.3 Bioactivity of Exos-AlgMA-MGs

**Fig. 3:**
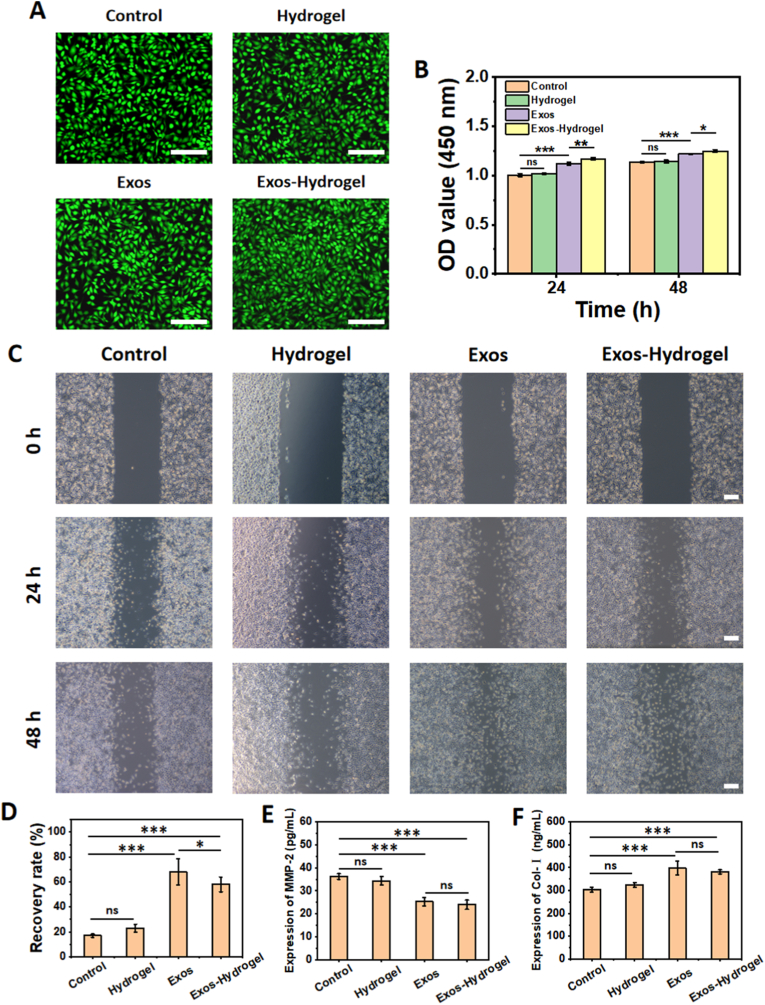
Effects of different treatments on fibroblast behaviors in vitro. (A) Live/Dead staining of fibroblasts in the control, hydrogel, exosomes (Exos), and exosomes-loaded hydrogel (Exos-Hydrogel) groups at 24 h. Scale bars: 200 μm. (B) CCK-8 absorbance of fibroblasts cultured with different treatments at 24 h and 48 h (n = 3; ns: no significance, ∗: p < 0.05, ∗∗: p < 0.01, ∗∗∗: p < 0.001). (C) Representative micrographs of scratch wounds at 0 h, 24 h, and 48 h for each treatment group. Scale bars: 200 μm. (D) Recovery rate of scratch assays at 48 h across treatment groups. (E, F) MMP-2 concentration and Col-I concentration in the culture medium of fibroblasts under different treatments. (n ≥ 5; ns: no significance, ∗: p < 0.05, ∗∗∗: p < 0.001).

The functional evolution of scleral fibroblasts is a central event in axial elongation and the development of myopia. Therefore, we first evaluated the effects of the AlgMA-MGs, exosomes, and Exos-AlgMA-MGs hydrogel on the proliferation and migration of fibroblasts. As shown in Fig. 3A and B, the cell viability and CCK-8 absorbance of fibroblasts treated with the hydrogel showed no significant difference from the control group at both 24 h and 48 h, indicating good cytocompatibility of the hydrogel. In contrast, fibroblasts treated with exosomes alone or the exosome-loaded hydrogel exhibited higher CCK-8 values at both time points, suggesting that both free exosomes and those released from the hydrogel enhanced cell proliferation. The scratch assay further confirmed the promotive effect of the exosome-loaded hydrogel on fibroblast migration. As shown in Fig. 3C and D, the wound recovery rate in the hydrogel-treated group showed no significant difference compared to the control group, reaching 17.0 ± 1.3% and 23.0 ± 3.2% after 48 h, respectively. In contrast, the exosome and exosome-loaded hydrogel treatment groups achieved significantly higher recovery rates of 68.1 ± 10.8% and 58.0 ± 5.8% at 48 h, demonstrating a marked enhancement in cell migration.

Scleral fibroblasts orchestrate extracellular matrix (ECM) homeostasis through collagen synthesis and the secretion of matrix metalloproteinases (e.g., MMP-2). In myopia progression, aberrantly elevated MMP activity disrupts this equilibrium, leading to collagen degradation, impairment of scleral structural integrity, and consequently, axial elongation. Based on this mechanistic understanding, we evaluated the ability of the exosome-loaded hydrogel to modulate fibroblast secretion of MMP-2 and collagen synthesis, aiming to correct the pathological scleral remodeling. As shown in Fig. 3E, ELISA quantification revealed that MMP-2 secretion levels in fibroblast cultures showed no statistically significant difference between the control group (36.2 ± 1.2 pg/mL) and the hydrogel-only group (34.3 ± 1.9 pg/mL). In contrast, administration of free exosomes significantly suppressed MMP-2 secretion to 25.3 ± 1.8 pg/mL. The exosome-loaded hydrogel demonstrated comparable efficacy, reducing MMP-2 concentration to 24.0 ± 2.0 pg/mL. Notably, both free exosomes and the exosome-loaded hydrogel markedly enhanced Col-I synthesis, with concentrations increasing from a baseline of 303.2 ± 10.3 ng/mL in the control group to 398.1 ± 30.3 ng/mL and 381.4 ± 9.3 ng/mL, respectively (Fig. 3F). These results demonstrate that the exosome-loaded hydrogel not only enhances fibroblast function by promoting proliferation and migration but also suppresses myopia progression via modulation of the TGF-β/MMP axis [38,39].

### 3.4 In vivo assessment of exosome-loaded hydrogel in the myopia model

**Fig. 4:**
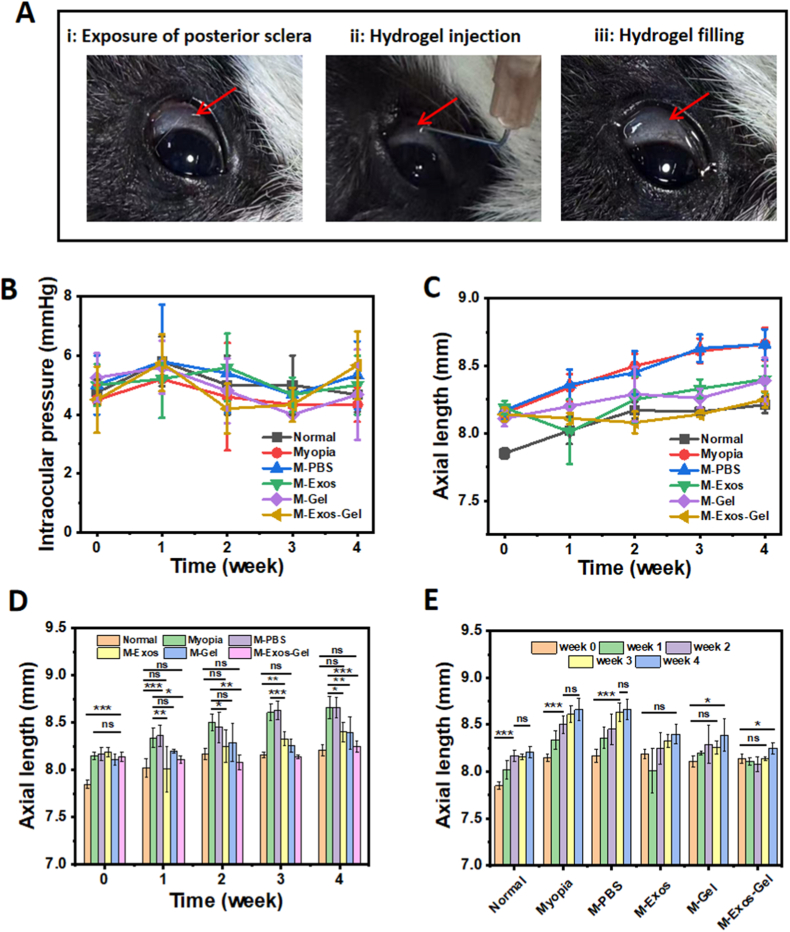
Hydrogel implantation procedure and longitudinal ocular parameters of the different treatment groups. (A) Sub-Tenon's implantation of the injectable exosome-loaded hydrogel at the posterior pole. (B) Temporal changes in intraocular pressure across the different treatment groups. (C) Temporal changes in axial length across the different treatment groups. (D) Axial length at each time point for the different treatment groups. (E) Axial length of each treatment group at different time points. (n ≥ 3; ns: no significance, ∗: p < 0.05, ∗∗: p < 0.01, ∗∗∗: p < 0.001).

A three-step posterior scleral delivery was established—(i) exposure of the posterior sclera, (ii) hydrogel injection, and (iii) posterior filling—demonstrating technical feasibility (Fig. 4A). Across 4-weeks, intraocular pressure (IOP) time-courses for all groups showed no sustained divergence from one another, indicating procedure- and material-level safety (Fig. 4B). As is shown in Fig. 4C–E, exosome-loaded hydrogel most strongly suppresses axial elongation. Longitudinal axial length (AL) traces diverged by treatment, which is reflected in cross-sectional biometry at week 2 and week 4 (Table 1 and Table 2). At week 2, AL (mean ± SD, mm) was: Normal 8.17 ± 0.06; Myopia 8.50 ± 0.09; M-PBS 8.45 ± 0.16; M-Exos 8.25 ± 0.17; M-Gel 8.29 ± 0.20; and M-Exos-Gel 8.08 ± 0.08. Relative to Myopia, the largest AL reduction was observed with M-Exos-Gel (Δ = 0.42 mm), followed by M-Exos (Δ = 0.25 mm) and M-Gel (Δ = 0.21 mm); M-PBS was close to Myopia (Δ = 0.05 mm). At week 4, AL was: Normal 8.21 ± 0.06; Myopia 8.66 ± 0.12; M-PBS 8.66 ± 0.11; M-Exos 8.40 ± 0.10; M-Gel 8.39 ± 0.17; and M-Exos-Gel 8.25 ± 0.06. The results showed the same trend as at week 2, M-Exos-Gel showed the greatest attenuation of axial elongation vs Myopia (Δ = 0.41 mm), with additional reductions for M-Gel (Δ = 0.27 mm) and M-Exos (Δ = 0.26 mm), while M-PBS matched Myopia (Δ = 0.00 mm). (Fig. 4C–E; Table 1, Table 2). The significant differences (p-values) in axial length among all treatment groups at each time point from week 1 to week 4 are shown in Table S1.

**Table 1:** Values of axial length and vitreous cavity depth of different treatment groups at week 2.

Ocular Component	Group	Changes in Right eyes at week 2, mm, Mean ± SD	P Value (Compared to Myopic group)
Axial length:anterior chamber +lens + vitreous chamber	Normal	8.17 ± 0.06	∗∗
	Myopia	8.50 ± 0.09	-
	M-PBS	8.45 ± 0.16	ns
	M-Exos	8.25 ± 0.17	∗
	M-gel	8.29 ± 0.20	ns
	M-Exos-gel	8.08 ± 0.08	∗∗
Vitreous chamber depth	Normal	3.47 ± 0.03	ns
	Myopia	3.53 ± 0.12	-
	M-PBS	3.50 ± 0.04	ns
	M-Exos	3.53 ± 0.09	ns
	M-gel	3.53 ± 0.16	ns
	M-Exos-gel	3.39 ± 0.07	ns

**Table 2:** Values of axial length and vitreous cavity depth of different treatment groups at week 4.

Ocular Component	Group	Changes in Right eyes at week 4, mm, Mean ± SD	P Value (Compared to Myopic group)
Axial length:anterior chamber +lens + vitreous chamber	Normal	8.21 ± 0.06	∗∗∗
	Myopia	8.66 ± 0.12	-
	M-PBS	8.66 ± 0.11	ns
	M-Exos	8.40 ± 0.10	∗
	M-gel	8.39 ± 0.17	∗∗
	M-Exos-gel	8.25 ± 0.06	∗∗∗
Vitreous chamber depth	Normal	3.45 ± 0.02	ns
	Myopia	3.59 ± 0.07	-
	M-PBS	3.63 ± 0.03	ns
	M-Exos	3.56 ± 0.15	ns
	M-gel	3.56 ± 0.13	ns
	M-Exos-gel	3.42 ± 0.05	ns

Therapeutically, treatment arms diverged in a biologically plausible hierarchy: exosome-loaded hydrogel (M-Exos-Gel) > hydrogel alone (M-Gel) ≈ exosomes alone (M-Exos) ≫ PBS ≈ myopia model. By week 2—and maintained at week 4—M-Exos-Gel showed the largest attenuation of axial elongation (ΔAL vs Myopia: −0.42 mm at week 2; −0.41 mm at week 4), bringing AL close to Normal at week 4 (8.25 vs 8.21 mm). The similar but smaller effects of M-Gel (mechanical support) and M-Exos (biologic modulation) indicate that each component contributes independently, while the superior performance of M-Exos-Gel suggests synergy—likely from posterior mechanical buttressing plus localized, sustained exosomal signaling that promotes collagen deposition and scleral matrix stabilization. The PBS group tracking the Myopia group confirms that the injection procedure has negligible impact.

These effects are consistent with a mechanism in which mechanical support reduces posterior wall strain and tangential stress, while exosomes enhance collagen I production and counter pathologic remodeling, yielding thicker, stiffer sclera and partial normalization of globe geometry. Notably, the durability of the effect through 4 weeks implies that residence time and on-target exposure matter; embedding exosomes in hydrogel likely improves both.

Vitreous chamber depth (VCD) trends mirror the axial-length effects, as shown in Table 1, Table 2. At week 2, VCD (mm) was: Normal 3.47 ± 0.03; Myopia 3.53 ± 0.12; M-PBS 3.50 ± 0.04; M-Exos 3.53 ± 0.09; M-Gel 3.53 ± 0.16; and M-Exos-Gel 3.39 ± 0.07. The M-Exos-Gel group showed the largest reduction vs Myopia (Δ = 0.14 mm). At week 4, VCD was: Normal 3.45 ± 0.02; Myopia 3.59 ± 0.07; M-PBS 3.63 ± 0.03; M-Exos 3.56 ± 0.15; M-Gel 3.56 ± 0.13; and M-Exos-Gel 3.42 ± 0.05. The M-Exos-Gel group again exhibited the lowest VCD (Δ = 0.17 mm vs Myopia), with other treated groups being closer to Myopia (−0.04 mm to 0.14 mm differences).

**Fig. 5:**
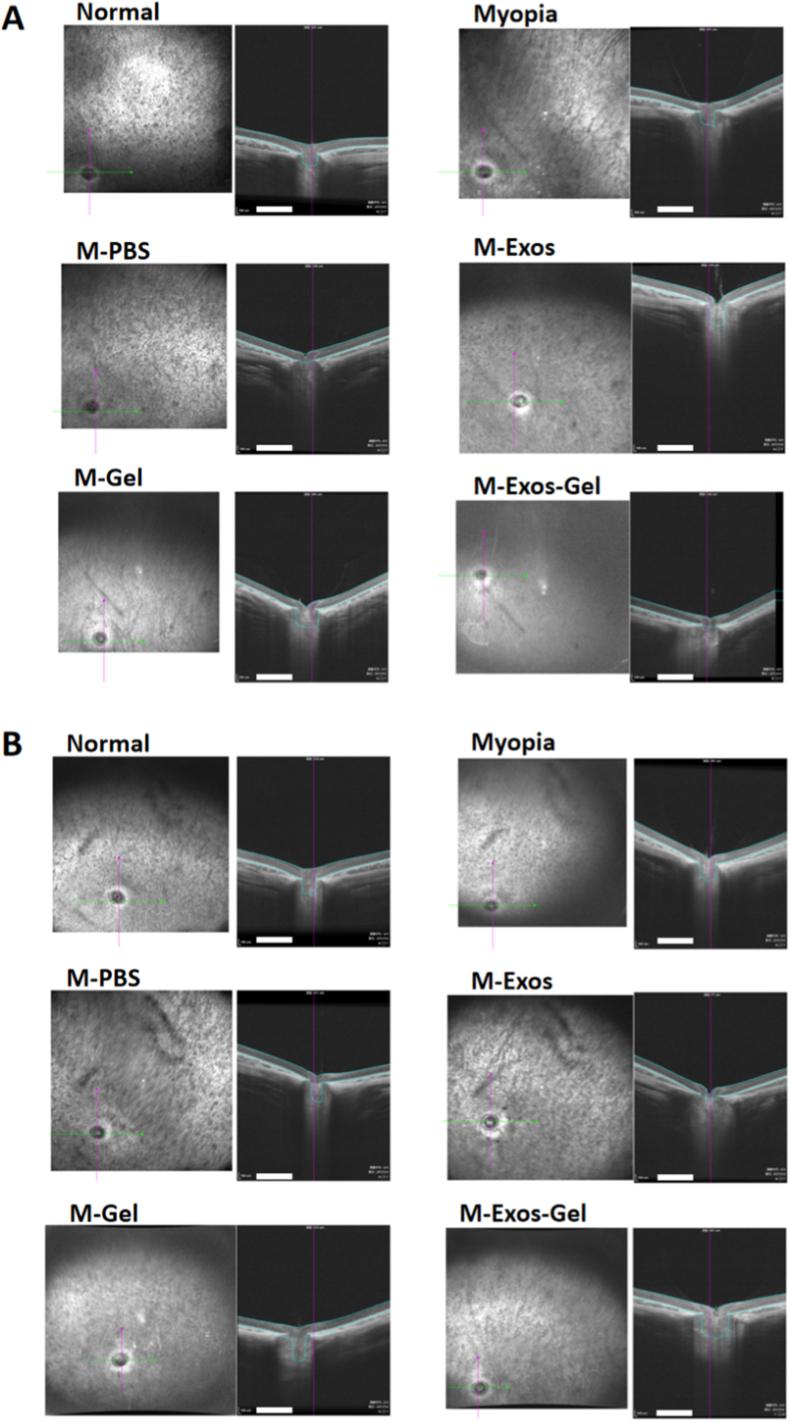
Retinal OCT images at week 2 (A) and week 4 (B) across the different treatment groups. Scale bars: 500 μm.

OCT shows structural preservation of the retina in Fig. 5. Representative OCT B-scans at week 2 and week 4 illustrate better lamination in treated eyes. Quantitatively, retinal thickness measurements at week 2 were as follows: 147.21 ± 1.01 μm in the normal group, 137.22 ± 1.45 μm in the myopia group, 131.85 ± 2.13 μm in the M-PBS group, 140.01 ± 1.90 μm in the M-Exos group, 124.71 ± 2.11 μm in the M-Gel group, and 146.46 ± 2.36 μm in the M-Exos-Gel group. By week 4, retinal thickness values were 143.52 ± 6.08 μm in the normal group, 128.89 ± 7.35 μm in the myopia group, 125.87 ± 4.29 μm in the M-PBS group, 130.71 ± 3.17 μm in the M-Exos group, 129.99 ± 6.44 μm in the M-Gel group, and 151.19 ± 1.68 μm in the M-Exos-Gel group (Table 3).

**Table 3:** Values of retinal thickness of different treatment groups at week 2 and week 4.

Ocular Component	Group	Changes in Right eyes at week 2, μm, Mean	Changes in Right eyes at week 4, μm, Mean
Retinal thickness	Normal	147.21 ± 1.01	143.52 ± 6.08
	Myopia	137.22 ± 1.45	128.89 ± 7.35
	M-PBS	131.85 ± 2.13	125.87 ± 4.29
	M-Exos	140.01 ± 1.90	130.71 ± 3.17
	M-gel	124.71 ± 2.11	129.99 ± 6.44
	M-Exos-gel	146.46 ± 2.36	151.19 ± 1.68

### 3.5 Histological analysis of sclera

**Fig. 6:**
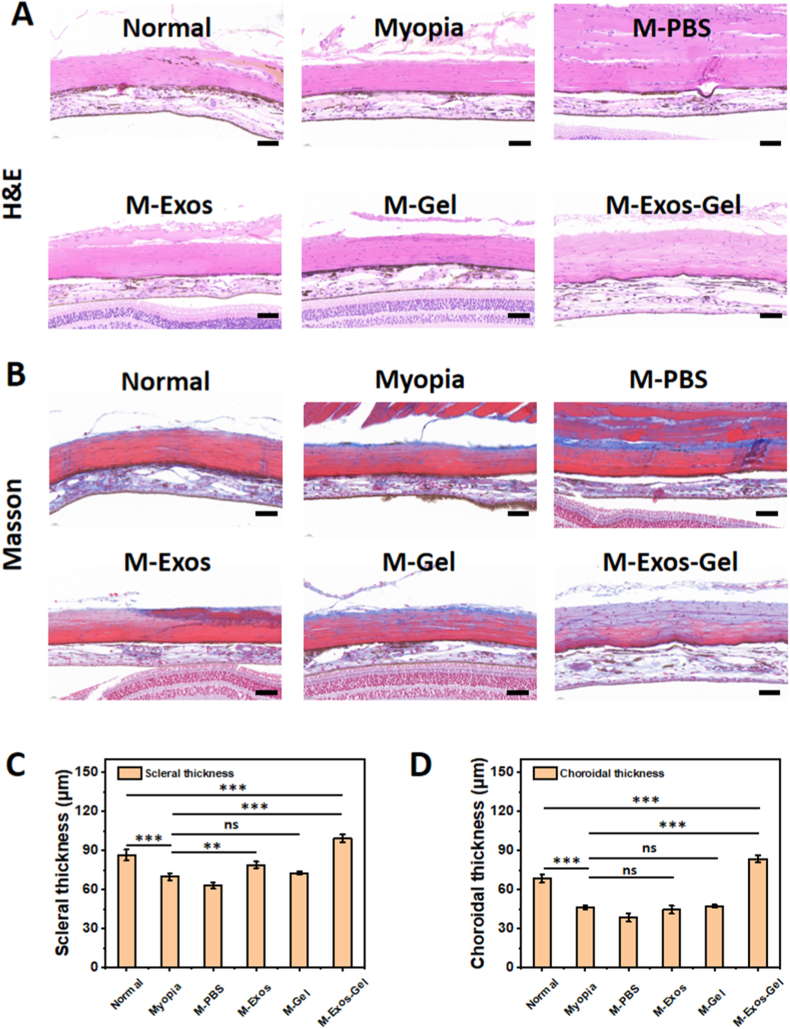
Histological analysis of the sclera–choroid across the different treatment groups at week 4. (A) H&E staining and (B) Masson's trichrome staining of the sclera–choroid. Scale bars: 50 μm. Quantitative analysis of (C) Scleral thickness and (D) Choroidal thickness. (n = 4; ns: no significance, ∗∗: p < 0.01, ∗∗∗: p < 0.001).

Sclera–choroid histology results support anti-myopic remodeling assumption. H&E and Masson's trichrome staining revealed qualitative differences across groups (Fig. 6A and B). Eyes receiving M-Exos-Gel showed visibly denser collagen bands in the posterior sclera on Masson staining and more compact scleral lamellae on H&E, consistent with reduced scleral creep. Quantitatively, scleral thickness followed the expected hierarchy (Fig. 6C): M-Exos-Gel > M-Gel ≈ M-Exos > M-PBS ≈ Myopia, with the combination group significantly thicker than Myopia and PBS and trending toward Normal. Choroidal thickness showed a similar but smaller pattern (Fig. 6D), with the greatest recovery in M-Exos-Gel and modest gains in the single-component groups, while M-PBS overlapped the Myopia group. These findings support a dual mechanism: hydrogel provides immediate posterior wall support (reducing local strain), while exosomes promote collagen deposition and matrix organization, together yielding a thicker, structurally more coherent sclera and partial choroidal recovery. The superiority of the combination group over either component alone is consistent with synergy between mechanical buttressing and localized, sustained exosomal signaling.

**Fig. 7:**
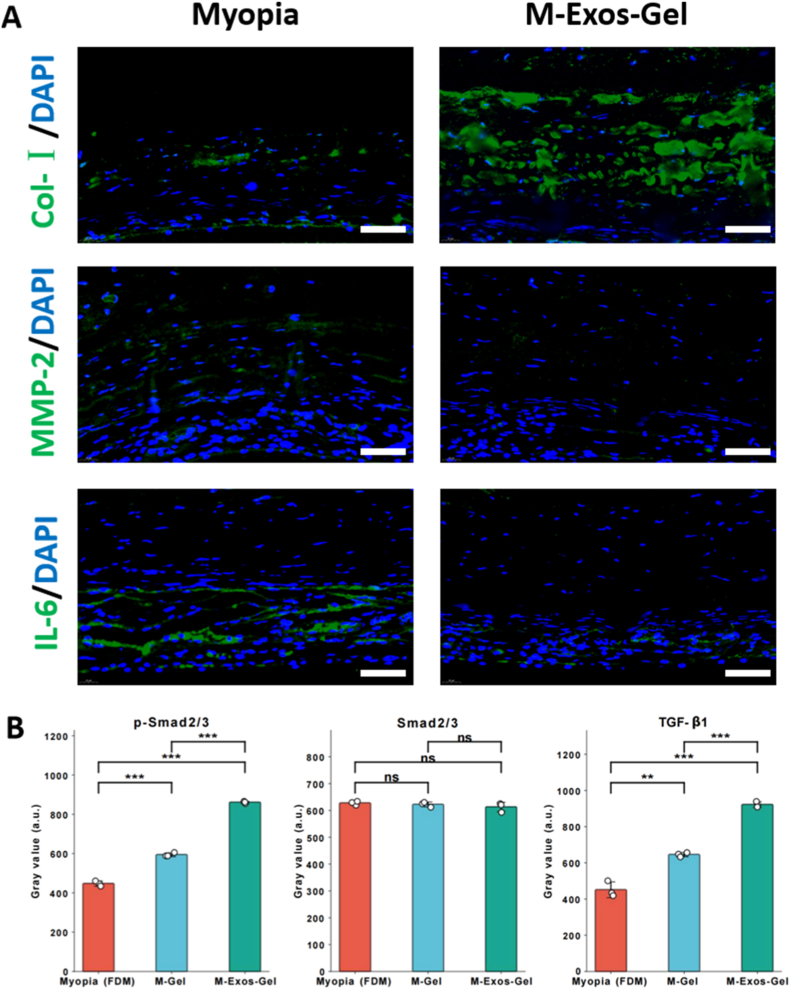
(A) Immunofluorescence staining for Col-Ⅰ, MMP-2, and IL-6 in the myopia group and M-Exos-Gel group at week 4. Scale bars: 50 μm. (B) Western blot analysis of TGF-β/Smad signaling in scleral tissue at week 4. (n = 3; ns: no significance, ∗∗: p < 0.01, ∗∗∗: p < 0.001).

Immunofluorescence images contrasted the Myopia model with M-Exos-Gel treatment, showing distinct patterns of signal intensity and localization, consistent with treatment-related modulation at the molecular level. The results showed reduced MMP-2 and IL-6, but enhanced Col-I signals in the posterior sclera in the M-Exos-Gel group (Fig. 7A). This shift suggests that exosomes promote a more fibrogenic microenvironment conducive to extracellular matrix preservation. Immunofluorescence provides a plausible mechanistic link between myopia and Exos-Gel. Relative to Myopia, Exos-Gel increases collagen-I signal while reducing MMP-2 and IL-6, suggesting a shift from matrix catabolism and para-inflammation toward a pro-matrix, anti-inflammatory state [40]. Such rebalancing of the TGF-β/MMP axis and cytokine milieu would be expected to strengthen scleral ECM, lower posterior wall strain, and thereby attenuate axial elongation—consistent with the biometric results [41]. The hydrogel likely enhances these effects by localizing and sustaining exosome exposure at the posterior pole, while simultaneously providing immediate mechanical support.

To further verify the involvement of the TGF-β signaling pathway in vivo, Western blot analysis was performed on scleral tissues (Fig. 7B). Compared with the Myopia group, the Exos-AlgMA-MG group exhibited significantly elevated levels of TGF-β1 and phosphorylated Smad2/3, while total Smad2/3 protein levels remained unchanged. These findings indicate activation of the canonical TGF-β/Smad2/3 signaling pathway in the sclera following exosome-loaded hydrogel treatment. Together with the immunofluorescence changes in Col-I and MMP-2, these data support the involvement of TGF-β/Smad signaling in treatment-associated ECM remodeling.

Notably, reduced TGF-β signaling in myopic eyes is known to attenuate collagen synthesis, while excessive MMP-2 activity drives collagen degradation and scleral weakening [42]. By elevating TGF-β signaling and concurrently suppressing MMP-2, MSC-Exos likely enhance collagen production and curtail collagen breakdown, thereby shifting the remodeling balance toward matrix reinforcement [43]. Our histological analyses support this mechanism: exosome-treated sclerae showed increased type I collagen deposition and more organized collagen fiber architecture relative to untreated myopic controls. By restoring collagen content and scleral structural integrity, MSC-Exos may contribute to reinforcement of the sclera and help resist the excessive axial elongation characteristic of progressive myopia.

## 4 Discussion

The injectable hydrogel system in our study played a critical role in enhancing the therapeutic efficacy of the exosomes by enabling sustained local delivery. Direct sub-Tenon injection of soluble factors typically suffers from rapid dispersal and clearance, resulting in a short residence time at the target tissue [44]. In contrast, the hydrogel forms an in situ depot that retains the exosomes at the posterior sclera and releases them gradually. This controlled release not only prolongs the presence of bioactive exosomes in the local microenvironment but also helps maintain therapeutic concentrations over time. Consistent with this, the MSC-Exos–loaded hydrogel produced a more pronounced inhibition of axial elongation in the myopic eyes compared to either exosomes or hydrogel alone, indicating a synergistic benefit of sustained delivery. By providing both a mechanical support and a slow-release vehicle, the hydrogel maximizes the interaction between exosomal factors and scleral cells, thereby potentially enhancing remodeling-related signaling.

Bone marrow-derived MSC exosomes were selected in this study because of their well-characterized paracrine activity, robust scalability, and reproducibility across batches, which are critical considerations for translational applications. Although scleral cell-derived exosomes may offer tissue-specific signaling advantages, their use is currently constrained by challenges in cell sourcing, expansion efficiency, and standardization. In this proof-of-concept study, MSC-derived exosomes provided a practical and biologically active platform to evaluate the feasibility of combining localized biomechanical support with exosome-mediated biological regulation.

A key strength of this approach is exosome–hydrogel therapeutic platform, which offers notable practical advantages due to its injectable, cell-free design. Unlike traditional scleral reinforcement interventions that involve graft implantation or other invasive procedures, our approach requires only a minimally invasive injection to deploy the material at the posterior pole. The hydrogel conforms to the scleral space and provides support without the need for sutures or large incisions, thereby reducing the risks associated with surgery. Moreover, the biomaterial is engineered to be biocompatible and biodegradable, meaning it can safely reside in the tissue and gradually dissolve without inciting significant inflammation. From a translational perspective, the use of acellular exosomes instead of live cells simplifies the therapy and improves safety: exosomes are intrinsically less immunogenic than their parent cells, and they can be produced and stored in a standardized manner. These features make repeat treatments feasible and enhance the scalability of the approach. Overall, the injectable MSC-Exos hydrogel represents a promising new paradigm for myopia control. It strengthens the sclera biomechanically while simultaneously modulating the underlying biological remodeling processes. With further development, this strategy could complement existing interventions (such as pharmacological or optical therapies) and provide an innovative option for patients at risk of high myopia and its sight-threatening complications.

Clinically, posterior scleral reinforcement has traditionally relied on mechanical stabilization to counteract axial elongation, with limited influence on the underlying biological processes of scleral remodeling. Progressive scleral thinning is a hallmark of myopia-associated scleral remodeling and is closely linked to increased susceptibility to axial elongation. Reduced scleral thickness reflects alterations in extracellular matrix organization and biomechanical integrity, which collectively lower resistance to intraocular pressure-driven expansion. In this context, strategies that preserve or restore scleral thickness may contribute to stabilization of axial length by enhancing both structural strength and biological homeostasis of the sclera. The dual-mechanism strategy presented here integrates immediate biomechanical buffering with sustained exosome-mediated biological regulation, offering a more comprehensive approach to myopia control. This localized and minimally invasive platform may improve both efficacy and safety compared with conventional reinforcement procedures. Compared with hydrogel-only or exosome-only interventions, the Exos-AlgMA-MG system demonstrated superior efficacy, suggesting a synergistic interaction between biomechanical reinforcement and biological regulation. This dual-mechanism strategy distinguishes the present approach from other emerging biomaterial-based or pharmacological strategies for myopia control, which typically target either mechanical or molecular pathways alone.

However, this study has several limitations. First of all, the therapeutic findings were demonstrated in an experimental guinea pig model of myopia, which may not fully capture the complexity of human myopic scleral remodeling. Caution is warranted in extrapolating these results to clinical practice, as inter-species differences in scleral composition, ocular size, and immune responses could influence treatment outcomes. Second, although the 4-week observation period allowed evaluation of early therapeutic effects, longer-term studies will be necessary to assess the durability of this intervention in the context of chronic myopia progression. Although this study focused on a single-dose administration to assess material retention and sustained biological activity, repeated or staged dosing strategies may be explored in future investigations. Progressive myopia in humans spans years, thus longer-term studies (in animal models or clinical trials) are needed to confirm sustained efficacy and safety. In addition, the in vivo release kinetics of exosomes from the hydrogel were not directly measured. While the hydrogel is presumed to provide controlled release, quantitative data on the release rate and duration would help to optimize dosing schedules and ensure consistent therapeutic levels. Addressing these limitations in future work will be important for translating the MSC-Exos hydrogel strategy toward human myopia management.

## 5 Conclusion

In conclusion, posterior-scleral delivery of Exos-AlgMA-MGs hydrogel is feasible and well tolerated, and it showed the greatest attenuation of axial elongation and VCD enlargement while better preserving retinal thickness than control conditions. The multi-scalereadouts—biometry, OCT, histology, and immunofluorescence— converge to support a protective effect against myopia-associated axial elongation of exosome-loaded hydrogel.

## CRediT authorship contribution statement

**Jingwen Hui:** Conceptualization, Data curation, Formal analysis, Funding acquisition, Investigation, Methodology, Project administration, Software, Supervision, Writing – original draft, Writing – review & editing. **Xiongfeng Nie:** Conceptualization, Data curation, Investigation, Methodology, Resources, Software, Writing – original draft, Writing – review & editing. **Zheya Han:** Investigation, Methodology. **Yuhua Rui:** Data curation, Methodology, Software. **Yuxi Bai:** Methodology. **Jingxuan Geng:** Methodology. **Wenguang Liu:** Conceptualization, Project administration, Supervision, Writing – review & editing. **Quanhong Han:** Conceptualization, Data curation, Formal analysis, Funding acquisition, Investigation, Methodology, Project administration, Supervision, Writing – review & editing.

## Declaration of competing interest

No conflict of interest exists.

We wish to confirm that there are no known conflicts of interest associated with this publication and there has been no significant financial support for this work that could have influenced its outcome.

## Data Availability

Data will be made available on request.
